# Randomised phase-II trial of CAPIRI (capecitabine, irinotecan) plus bevacizumab *vs* FOLFIRI (folinic acid, 5-fluorouracil, irinotecan) plus bevacizumab as first-line treatment of patients with unresectable/metastatic colorectal cancer (mCRC)

**DOI:** 10.1038/bjc.2011.594

**Published:** 2012-01-12

**Authors:** J Souglakos, N Ziras, S Kakolyris, I Boukovinas, N Kentepozidis, P Makrantonakis, S Xynogalos, Ch Christophyllakis, Ch Kouroussis, L Vamvakas, V Georgoulias, A Polyzos

**Affiliations:** 1 Hellenic Oncology Research Group (HORG), 55 Lomvardou str., Athens 11470, Greece

**Keywords:** CAPIRI, FOLFIRI, bevacizumab, mCRC, phase-II trial

## Abstract

**Background:**

To compare the efficacy and safety of CAPIRI+bevacizumab (Bev) in comparison with FOLFIRI+Bev as first-line treatment for patients with metastatic colorectal cancer (mCRC).

**Methods:**

Patients were randomised to receive either FOLFIRI plus Bev 5 mg kg^−1^ every 2 weeks (Arm-A) or CAPIRI plus Bev 7.5 mg kg^−1^ every 3 weeks (Arm-B).

**Results:**

Three hundred thirty-three patients (Arm-A=167; Arm-B=166) were enrolled into the study. No difference was observed in median progression-free survival (PFS) (10.0 and 8.9 months; *P*=0.64), overall survival (25.7 and 27.5 months; *P*=0.55) or response rates (45.5 and 39.8.7% *P*=0.32) for FOLFIRI-Bev and CAPIRI-Bev, respectively. Patients treated with CAPIRI-Bev presented significantly higher incidence of diarrhoea (*P*=0.005), febrile neutropenia (*P*=0.003) and hand–foot skin reactions (*P*=0.02) compared with patients treated with FOLFIRI-Bev. Treatment delays (*P*=0.05), dose reduction (*P*<0.001) and treatment discontinuation owing to toxicity (*P*=0.01) occurred more frequently in the CAPIRI-Bev arm.

**Conclusion:**

The PFS of FOLFIRI-BEV is not superior to that observed with the CAPIRI-Bev regimen. CAPIRI-Bev has a less favourable toxicity profile, requiring dose reductions, in order to be considered as an option in first-line treatment of patients with mCRC.

Despite the improvement in median overall survival (mOS) over the last 10 years, metastatic colorectal cancer (mCRC) remains a major public health problem accounting for 8% of cancer deaths in adults in the western world (Jemal *et al*, 2011). Combination chemotherapy with infusional 5-fluorouracil (FU) and folinic acid (FA) with either irinotecan (FOLFIRI) or oxaliplatin (FOLFOX) is commonly used in the daily practice. Expansion of mOS and 5-year survival rate have been correlated with the proportion of patients receiving all active chemotherapeutic agents (Grothey *et al*, 2004) and the increasing use of hepatic or/and pulmonary resection of metastatic lesions (Kopetz *et al*, 2009).

The addition of bevacizumab (Bev), a monoclonal antibody targeting vascular endothelial growth factor (VEGF), to combination chemotherapy in the first-line setting appears to increase the efficacy of systemic treatment in randomised trial (progression-free survival (PFS) and/or mOS) (Hurwitz *et al*, 2004; Saltz *et al*, 2008), but the magnitude of the benefit is debatable. However, one can argue that the benefit from addition of Bev to irinotecan-based chemotherapy (Hurwitz *et al*, 2004) is generally greater than that observed when it is combined with oxaliplatin-based regimens (Saltz *et al*, 2008).

Capecitabine, an oral fluoropyrimidine, was designed to mimic continuous infusion 5-FU and to generate 5-FU preferentially in the tumour tissue (Miwa *et al*, 1998; Schuller *et al*, 2000). Capecitabine has similar efficacy compared with 5-FU/LV as first-line treatment in mCRC patients (Van *et al*, 2000); the advantage of capecitabine is its convenient oral administration (Scheithauer *et al*, 2003). Capecitabine in combination with oxaliplatin (CAPOX) has consistently demonstrated similar efficacy results compared with the FOLFOX regimen (Cassidy *et al*, 2008). Likewise, the combination of capecitabine with irinotecan (CAPIRI) was proven effective and safe in a large randomised trial (Koopman *et al*, 2007). Finally, the combination of Bev and capecitabine has a synergistic effect, in an *in vivo* xenograft model, with a greater duration of tumour growth inhibition than with either agent alone (Kolinsky *et al*, 2009).

Based on these data, the Hellenic Oncology Research Group (HORG) designed a randomised phase-II trial in order to investigate the efficacy and safety of addition of Bev to FOLFIRI or CAPIRI as front-line treatment of patients with mCRC.

## Patients and methods

### Eligibility criteria

Untreated patients with mCRC were eligible for the study. Patients who had received prior adjuvant chemotherapy with fluoropyrimidines±oxaliplatin were eligible if they had remained free of disease for at least 6 months after completion of treatment. Other eligibility criteria were as follows: age ⩾18 years; performance status (ECOG) 0–2; at least one measurable lesion according to the Response Evaluation Criteria in Solid Tumors (RECIST) criteria (Therasse *et al*, 2000); adequate haematologic parameters (absolute neutrophil count ⩾1.5 × 10^9^/l and platelets ⩾100 × 10^9^/l); creatinine and total bilirubin ⩽1.25 times the upper limit of normal; aspartate and alanine aminotransferases ⩽3.0 times the upper limit of normal; absence of active infection or malnutrition; and absence of a second primary tumour except a skin squamous carcinoma or an *in situ* carcinoma of the uterine cervix. Patients with liver metastases involving more than 50% of the liver parenchyma; chronic diarrhoea; myocardial infarction within 1 year before treatment initiation; stroke; pre-existing bleeding diatheses or coagulopathy, or need for full-dose anticoagulation therapy or history of deep vein thrombosis within 6 months prior to registration; uncontrolled hypertension; pre-treatment proteinuria ⩾ grade-2; and central nervous system metastases were excluded.

The study was approved by the Ethics and Scientific Committees of each participating centre and all patients gave written informed consent prior to study enrolment.

### Treatment protocol

Patients were randomised to receive either FOLFIRI-Bev (Arm-A: irinotecan at the dose of 180 mg m^−2^, iv, on day 1; FA at the dose of 200 mg m^−2^, iv, on days 1 and 2; and 5-FU at the dose of 400 mg m^−2^day^−1^, iv, bolus and 600 mg m^−2^ day^−1^, as a 22-h iv continuous infusion, on days 1 and 2, plus 5 mg kg^−1^ Bev on day 1, every 2 weeks) or CAPIRI-Bev (Arm-B: capecitabine at the dose of 2000 mg m^−2^, p.o., on days 1–14; irinotecan at the dose of 250 mg m^−2^, iv, on day 1; and Bev at the dose of 7.5 mg kg^−1^, iv, every 3 weeks). Stratification factors were age (⩽65 years *vs* >65 years), extent of metastatic disease (liver limited *vs* other) and prior adjuvant chemotherapy (yes *vs* no). Routine antiemetic prophylaxis with a 5-hydroxytryptamine-3-receptor antagonist was used in both arms. Treatment was administered until disease progression or unacceptable toxicity, or consent withdrawal.

Patients were assessed for toxicity before each cycle using the National Cancer Institute Common Toxicity Criteria version 3.0. Chemotherapy was delayed until recovery if neutrophils were less than 1.5 × 10^9^/l or platelets less than 100 × 10^9^/l, or for significant (more than grade-II) persisting non-haematologic toxicity.

Doses of all drugs were reduced by 15% in subsequent cycles in case of grade-4 neutropenia or grade-3–4 thrombocytopenia lasting for more than 3 days, or in case of febrile neutropenia. No prophylactic administration of granulocyte colony-stimulating factor was allowed. Doses of irinotecan and 5-FU or capecitabine were reduced by 15% in subsequent cycles in case of grade-3 or 4 diarrhoea. The 5-FU or capecitabine dose was reduced in case of grade-3–4 stomatitis or dermatitis. Bevacizumab was permanently discontinued in patients developing gastrointestinal perforation, wound dehiscence requiring medical intervention, serious bleeding, nephrotic syndrome or hypertensive crisis. Temporary discontinuation of Bev administration was implemented in patients with evidence of moderate-to-severe proteinuria and in patients with severe hypertension that was not controlled with medical management.

### Patient evaluation

Pre-treatment evaluation included medical history and physical examination, complete blood cell count (CBC) with differential and platelet count, blood chemistry, serum levels of carcinoembryonic antigen, and computed tomographic (CT) scans of the chest and imaging of the abdomen (CT or MRI). Pre-treatment evaluation had to be performed within 2 weeks prior to study entry. During treatment, a CBC with was performed weekly. In addition, patients were clinically assessed and blood chemistry was performed before each treatment cycle. Response to treatment was evaluated every 8 weeks according to the RECIST criteria (Therasse *et al*, 2000) in order to gain comparable efficacy results between the two treatment regimens.

### Statistical considerations

The primary endpoint of the study was PFS. Secondary endpoints were mOS, response to treatment and safety profile in terms of adverse events incidence, dose reductions and treatment delays. Based on the results of the BICC trial (Fuchs *et al*, 2007) the study was designed in order to detect a 3-month difference (8 *vs* 11 months) in PFS with an 80% power at a significance level of 0.05. In order to achieve the statistical hypothesis, 165 patients (per arm) should be enrolled in 36 months, with an additional follow-up period of 24 months.

The Kaplan–Meier method was used to estimate PFS and survival curves, and log-rank test was used to compare curves. Cox proportional hazards modelling was used to calculate hazard ratios (HRs) and confidence intervals (CIs). Heterogeneity tests were performed in order to determine whether the effect size for the subgroups varies significantly from the main effect. Forest plots were used in order to investigate the effect of the studied variables apart in accordance to the overall effect for each case. *χ*^2^-Tests were used to compare toxicity and confirmed response rates. *P*-values less than 0.05 were considered statistically significant for all comparisons. Progression-free survival was defined as the interval from the time of enrolment to the date of first documented disease progression or patient's death from any cause. Overall survival is considered the time interval from the date of enrolment until the date of death from any cause. The duration of response was measured from the first documentation of response to disease progression.

## Results

### Patients’ characteristics

**Figure 1:**
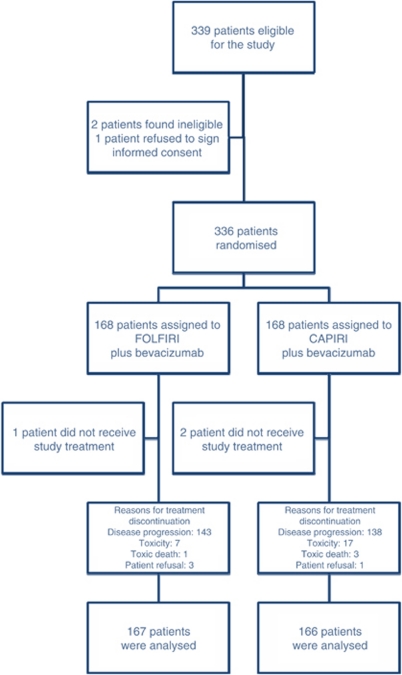
CONSORT diagram of the study.

From June 2005 to June 2008, 336 patients with unresectable mCRC were enrolled into the study at 23 institutions throughout Greece. Two patients, in the CAPIRI+Bev Arm-B and one patient in the FOLFIRI+Bev Arm-A received no study treatment because they were found ineligible. The remained 333 were randomly allocated to receive front-line treatment and received at least one chemotherapy cycle (167 in Arm-A and 166 in Arm-B), and were analysed for efficacy and safety (Figure 1). Patients’ characteristics were typical for mCRC in the western world (Table 1). More specifically, about one half of patients in both arms were >65 years old, the vast majority (97% in Arm-A and 98% in Arm-B) had PS of 0–1, one-third had received prior adjuvant chemotherapy, whereas 37% in Arm-A and 38% in Arm-B had metastatic disease limited to the liver. Similarly, in one-third of patients in each arm, metastases were synchronous to diagnosis of the primary tumour. Overall, 25% and 22% of the patients in Arm-A and Arm-B, respectively, were classified as high risk according to the Kohne prognostic index (Kohne *et al*, 2002).

### Compliance with treatment

In the FOLFIRI-Bev arm, 1494 treatment cycles were administered compared with 871 cycles in the CAPIRI-Bev arm. The median number of cycles was 11 (range 1–20) and 6 (range 1–10) per patient treated with the FOLFIRI-Bev and CAPIRI-Bev regimen, respectively; however, the median treatment period was similar (5.5 months) for both arms.

Treatment delays were observed in 134 (9.0%) chemotherapy courses in the FOLFIRI-Bev arm and 136 (15.6%) in the CAPIRI-Bev arm (*P*=0.05); the median duration of delay was 4 days (range 1–14) in the FOLFIRI-Bev arm and 7 days (range 1–18) in the CAPIRI-Bev arm (*P*=0.23). In the FOLFIRI-Bev arm the reasons of delay were haematologic (*n*=60, 4.0%), non-haematologic (*n*=34, 2.2%) or combined (*n*=54, 3.6%) toxicity. In the CAPIRI-Bev arm treatment was delayed due to haematologic (*n*=44, 5.2%), non-haematologic (*n*=82, 9.4%) or combined (*n*=10, 1.1%) toxicity. The incidence of non-haematologic toxicity was significantly higher in the CAPIRI-Bev arm (*P*=0.031). The median interval between cycles was 14 (range 14–28) and 21 (range 21–39) days in the FOLFIRI-Bev and CAPIRI-Bev arms, respectively. Dose reduction was required in 65 (4.3%) cycles in the FOLFIRI-Bev arm and 95 (10.9%) cycles in the CAPIRI-Bev arm (*P*<0.001). The main reasons for dose reduction were haematologic (FOLFIRI-Bev (*n*=21, 1.4%) and CAPIRI-Bev (*n*=25, 2.8%)), non-haematologic (FOLFIRI-Bev (*n*=30, 2.0%) and CAPIRI-BEV (*n*=49, 5.6%)) or both (FOLFIRI-Bev (*n*=9, 0.6%) and CAPIRI-BEV (*n*=21, 2.4%)) toxicities. Treatment was discontinued in seven (4.2%) patients enrolled in the FOLFIRI-Bev arm and 17 (10.2%) in the CAPIRI-Bev arm (*P*=0.04). The delivered relative dose intensity was 90% for irinotecan, 92% for 5-FU/FA and 94% for Bev of the protocol-planned doses in the FOLFIRI-Bev arm, and 79% for irinotecan, 82% for capecitabine and 97% for Bev in the CAPIRI-Bev arm.

### Efficacy

**Figure 2:**
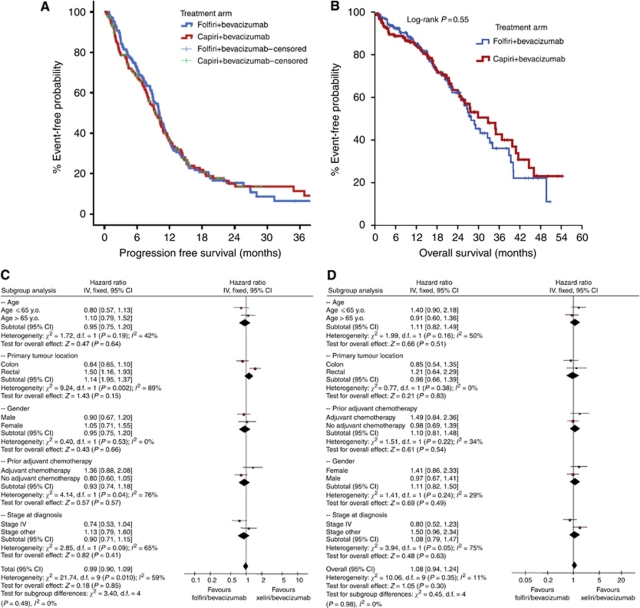
(**A**) Progression-free survival of patients treated with FOLFIRI+Bev or CAPIRI+Bev. (**B**) Overall survival of patients treated with FOLFIRI+Bev or CAPIRI+Bev. (**C**) Forest plots of PFS of patients treated with FOLFIRI+Bev or CAPIRI+Bev. (**D**) Forest plots of OS of patients treated with FOLFIRI+Bev or CAPIRI+Bev.

After a median follow-up period of 32 months (range 1–64 months), 143 (86%) patients in FOLFIRI-Bev and 138 (83%) in CAPIRI-Bev experienced disease progression, whereas 90 (54%) and 87 (52%) patients, respectively died. There was no statistical difference in terms of median PFS between the two arms: 10.0 months (95% CI: 8.9–11.1 months) for patients treated with FOLFIRI-Bev compared with 8.9 months (95% CI: 7.3–10.2 months) for those treated with CAPIRI-Bev (HR=0.99; 95% CI: 0.90–1.09; *P*=0.85; Figure 2A). Similarly, there was no statistical difference in terms of mOS between the two regimens (Figure 2B). Patients treated with the FOLFIRI-Bev regimen presented an mOS of 25.7 months (95% CI: 23.0–28.4 months) whereas those treated with CAPIRI-Bev showed an mOS of 27.5 months (95% CI: 22.6–32.3 months) (HR=1.08; 95% CI: 0.94–1.24; *P*=0.30). In addition, no difference in PFS (Figure 2C) or mOS (Figure 2D) has been observed in subgroup analysis.

In the ITT population, the overall response rate (ORR) was 45.5% (95% CI: 38.0–53.1%) in the FOLFIRI-Bev arm and 39.8% (95% CI: 32.3–47.2%) in the CAPIRI-Bev arm (*P*=0.32). More specifically, complete responses (CRs) were recorded in 11 (6.6%) and partial responses (PRs) in 65 (38.9%) patients treated with FOLFIRI-Bev, whereas 9 (6.5%) and 53 (31.9%) patients experienced CRs and PRs, respectively, in the CAPIRI-Bev arm. The median time of response duration was 8.2 (95% CI: 7.6–8.9) and 8.0 months (95% CI: 6.6–9.5) in the FOLFIRI-Bev and CAPIRI-Bev arm, respectively (*P*=0.58). Fifty (29.9%) patients treated with FOLFIRI-Bev and 52 (31.3%) patients treated with CAPIRI-Bev experienced stabilisation of disease, whereas 41 (24.6%) and 48 (28.9%) patients, respectively, had progression of their disease at the first efficacy evaluation. Secondary R0 metastasectomy was performed in six (3.6%) patients treated with FOLFIRI-Bev and three (1.8%) patients treated with CAPIRI-Bev (*P*=0.38). In patients with liver-limited disease, R0 resections were obtained in 5 (8%) and 3 (5%) patients in the FOLFIRI-Bev and the CAPIRI-Bev arm, respectively (*P*=0.88).

### Toxicity

Patients treated with CAPIRI-Bev had a significantly higher incidence of grade-3/4 febrile neutropenia (*P*<0.001), diarrhoea (*P*=0.003) and hand–foot skin reaction (*P*=0.03) compared with patients treated with FOLFIRI-Bev (Table 2). All other adverse events were equally distributed between the two treatment arms. Bevacizumab-related serious adverse events were rare in both arms. Grade-3/4 hypertension was observed in 3.8% and 4.2% of the patients in the FOLFIRI-Bev and CAPIRI-Bev arm, respectively. One patient in each arm presented with a large bowel perforation, which was lethal in one of them (in FOLFIRI-Bev arm). Three additional deaths, all due to febrile neutropenia combined with diarrhoea, occurred in the CAPIRI-Bev arm during treatment. The death rates within the first 60 days of treatment were 2.4% (95% CI: 1.0–4.1%) for patients treated with the FOLFIRI-Bev regimen and 4.1% (95% CI: 2.3–5.9%) for those treated with the CAPIRI-Bev regimen (*P*=0.42).

### Second-line treatment

Although that second-line treatments were not specified by the protocol, the regimens administered after disease progression were recorded. An oxaliplatin-based second-line treatment was administered in 76% and 72% of patients after progression to FOLFIRI-BEV or CAPIRI-BEV, respectively (Table 3). Cetuximab either as monotherapy or in combination with irinotecan was administered in 38% of the patients after progression to FOLFIRI-BEV and 30% of those with progression after CAPIRI-Bev. Bevacizumab administration was continued in the second-line setting in 23% of the patients in each arm (Table 4).

## Discussion

Addition of monoclonal antibodies targeting either VEGF or EGFR to irinotecan–5-FU/FA combination chemotherapy in some studies has demonstrated an increase in RR, PFS and mOS compared with chemotherapy alone (Hurwitz *et al*, 2004; Van *et al*, 2009). To the best of our knowledge the current study is the first randomised trial comparing the combination of Bev with the standard FOLFIRI regimen with its combination with the outpatient CAPIRI regimen. The primary endpoint, a 3-months increase in PFS, was not met as no statistically significant difference has been observed between the two treatment arms (HR=0.99; 95% CI: 0.90–1.09; *P*=0.85). Similarly, no differences have been observed in terms of mOS (HR=1.08; 95% CI: 0.94–1.24; *P*=0.30) and of ORR (*P*=0.32).

The efficacy parameters of FOLFIRI-Bev are in the same range with that reported in previous phase-II (Kopetz *et al*, 2010) and III trials (Fuchs *et al*, 2008), and compare favourably with those reported for FOLFIRI alone (Tournigand *et al*, 2004; Souglakos *et al*, 2006; Van *et al*, 2009). In addition, in the present study, the PFS and mOS for CAPIRI-Bev compare favourably with those reposted for CAPIRI alone (Bajetta *et al*, 2004; Borner *et al*, 2005; Koopman *et al*, 2007), and are in the same range with those recently reported in a phase-II study (Garcia-Alfonso *et al*, 2010). The CAPIRI regimen has proved its efficacy in several phase-II studies (Bajetta *et al*, 2004; Borner *et al*, 2005; Rea *et al*, 2005), and in a large randomised trial (Koopman *et al*, 2007) with PFS and mOS of 8.0 and 17.5 months, respectively. Despite that, major concerns regarding the efficacy of the regimen have been raised from the BICC-C trial (Fuchs *et al*, 2007). This trial reported that administration of CAPIRI led to significantly lower PFS (5.8 months) in comparison with FOLFIRI, whereas the mOS was comparable between the two arms. Overall, the results of the current investigation support that FOLFIRI-Bev and CAPIRI-Bev are equally effective in terms of ORR, PFS and mOS.

Overall survival was not the primary endpoint of the study as it is quite difficult to drawn conclusions from a randomised phase-II study. Taking into account these limitations, it is noteworthy that the mOS observed in the current study in both arms is one of the highest reported in randomised trials. Despite the fact that patients with massive liver infiltration from the tumour (>50% of the total parenchyma) or with central nervous system metastasis were excluded from the study, and that this may be considered as a selection bias, the long mOS observed in the current study could not be explained from the characteristics of the patients enrolled into the study. A significant proportion of patients had received prior adjuvant treatment and the percentage of patients with favourable characteristics, such as disease limited to the liver, low risk according to the Kohne index and metachronous metastatic disease, were in the same range with those recorded in other trials (Hurwitz *et al*, 2004; Van *et al*, 2009; Moosmann *et al*, 2011). The percentage of patients who underwent secondary resection was low (3.5% and 1.8% for FOLFIRI-Bev and CAPIRI-Bev, respectively) but in the same range with that observed in studies with combination of irinotecan plus fluoropyrimidines and Bev (Hurwitz *et al*, 2004). Thus, it seems difficult to explain of the high mOS observed in the current study. A significant proportion of patients received effective second-line treatment. Approximately 60% of patients were treated with oxaliplatin-based regimens, whereas monoclonal antibodies against EGFR were administered in >30% of the cases and Bev beyond progression in approximately one-quarter of the cases. It is generally accepted that the mOS is significantly correlated with the proportion of patients receiving all active chemotherapeutic agents over the disease course (Grothey *et al*, 2004), and that salvage treatment with anti-EGFR monoclonal antibodies could increase PFS and OS in KRAS wt patients (Amado *et al*, 2008; Karapetis *et al*, 2008). In addition, data from observational cohort studies support that use of Bev beyond progression could be associated with improved mOS (Grothey *et al*, 2008), although this point has not yet been investigated in prospective randomised trials.

The main difference recorded in the present study concerns the toxicity profile of the two regimens. Indeed, patients treated with CAPIRI-Bev had a significantly higher incidence of diarrhoea (*P*=0.005), febrile neutropenia (*P*=0.003) and hand–foot skin reactions (*P*=0.02) compared with patients treated with FOLFIRI-Bev. The timing of safety assessments was different between the two treatments arm (every 2 weeks in the FOLFIRI+Bev arm and every 3 weeks in the CAPIRI+Bev arm). Despite that, the differences in safety profile could not be explained from the dissimilarity in the timing of safety assessment as the higher toxicity grade was recorded in each assessment and the worst toxicity per patient is reported. Moreover, despite the fact that the delivered relative dose intensity was comparable between the two arms, treatment delays (*P*=0.05), dose reductions (*P*<0.001) and treatment discontinuation owing to toxicity (*P*=0.01) occurred more frequently in the CAPIRI-Bev arm. The incidence of grade-3/4 toxicities in the FOLFIRI-Bev was in the same range with those reported for FOLFIRI alone (Tournigand *et al*, 2004; Souglakos *et al*, 2006). The incidence of severe toxicities with CAPIRI-Bev was, also, comparable with those observed in the BICC-C and CAIRO studies (Fuchs *et al*, 2007; Koopman *et al*, 2007) for CAPIRI alone. The additional gastro-intestinal toxicity of the CAPIRI-Bev regimen observed in the current study should not be considered as specific to the combination of capecitabine with irinotecan. In fact, the incidence of severe diarrhoea is higher with the XELOX regimen in comparison with FOLFOX4 (Cassidy *et al*, 2008).

Overall, addition of Bev in either arm does not seem to increase the incidence of adverse events. Recently, data from studies investigating lower doses of CAPIRI in combination with either Bev (Garcia-Alfonso *et al*, 2010) or cetuximab (Moosmann *et al*, 2011) reported a more favourable toxicity profile with lower incidence of diarrhoea and neutropenia, as well as lower rates of dose reductions and treatments delays.

Overall, the results of the current study show that the CAPIRI-Bev regimen at the doses used in this study demonstrated comparable efficacy with FOLFIRI-Bev but with increased incidence of diarrhoea, neutropenia and hand–foot skin reactions. Owing to the increase toxicity and frequent dose modification, lower doses of cytotoxics would be considered in future trials using the CAPIRI-Bev regimen as front-line treatment for patients with mCRC.

## Figures and Tables

**Table 1:** Patients’ characteristics

	**FOLFIRI (*n*=167)**		**CAPIRI (*n*=166)**		
**Characteristics**	***n***	**%**	***n***	**%**	***P*-value**
*Age*					
Median (range)	66 (33–80)		67 (26–80)		
>65 years	90	54	91	55	0.22
					
*Gender*					
Male	104	62	109	66	0.56
Female	63	38	57	34	
					
*Performance status (ECOG)*					
0	52	31	49	30	0.88
1	110	66	113	68	
2	5	3	4	2	
					
*Primary tumour location*					
Colon	124	74	133	80	0.36
Rectum	43	26	33	20	
					
*Prior adjuvant chemotherapy*					
None	110	66	111	67	0.48
5-FU/LV	21	12	21	13	
Oxaliplatin 5-FU	36	22	34	20	
					
*Number of metastatic sites*					
1	81	49	82	49	0.61
⩾2	86	51	84	51	
Median (range)	2 (1–6)		2 (1–6)		
Liver-limited disease	62	37	63	38	0.84
					
*Metastases*					
Synchronous	56	34	54	33	0.89
Metachronous	111	66	112	67	
					
*Kohne prognostic index*					
Low-risk	53	32	56	34	0.31
Intermediate-risk	72	43	74	44	
High-risk	42	25	36	22	

**Table 2:** Treatment efficacy

	**FOLFIRI+Bev**	**XELIRI+Bev**	
**ITT population**	***n*=167**	***n*=166**	***P*-value**
Progression-free survival (months) (95% CI)	10 (8.9–11.1)	8.9 (7.3–10.2)	0.64
Median overall survival (months) (95% CI)	25.7 (23.0–28.4)	27.5 (22.6–32.3)	0.55
Response duration (months) (95% CI)	8.2 (7.6–8.9)	8.0 (6.6–9.5)	0.58
Response rate (%) (95% CI)	45.5 (38.0–53.1)	39.8 (32.3–47.2)	0.32
Disease control rate (%) (95% CI)	75.4 (66.3–84.8)	71.1 (64.7–82.1)	0.39
R0 resections (%)	3.6	1.8	0.38

Abbreviation: ITT=intent-to-treat.

**Table 3:** Incidence of common toxicities with the FOLFIRI+Bev and CAPIRI+BEV regimens (worst toxicity per patient)

	**FOLFIRI+Bev**	**CAPIRI+Bev**		**FOLFIRI+Bev**	**CAPIRI+Bev**	
	**Any grade**			**Grade-3/4**		
	***n***	**%**	***P*-value**	***n***	**%**	***P*-value**
*Adverse event*						
Any	90.1	93.2	0.78	30.6	37.4	0.19
Neutropenia	80.2	78.7	0.84	24.5	17.9	0.62
Febrile neutropenia	1.2	4.8	0.003	0.6	4.8	<0.001
Anaemia	64.1	62.0	0.89	0.6	1.2	0.29
Thrombocytopenia	20.3	31.2	0.57	0.6	0.6	0.91
Alopecia	52.4	60.7	0.72	11.2	18.4	0.35
Diarrhoea	48.6	64.6	0.005	9.2	15.8	0.003
Nausea	43.4	51.6	0.64	3.2	5.4	0.21
Mucositis	16.2	18.7	0.58	1.2	1.2	0.98
Hand–foot skin reaction	14.3	34.6	0.02	1.2	4.2	0.03
Fatigue	39.7	35.8	0.81	4.6	3.8	0.83
Hypertension	20.8	24.2	0.81	3.8	4.2	0.98
Bleeding	6.0	6.2	0.97	0	0	—
Perforation	0	0	—	0.6	0.6	1.0

**Table 4:** Therapies administered after progression to first-line treatment

	**FOLFIRI+Bev**		**CAPIRI+Bev**		
**Second-line treatment**	**No. of patients**	**%**	**No. of patients**	**%**	***P*-value**
Any	128	76	122	72	0.41
LOHP-based	104	62	98	59	0.39
Irinotecan-based	24	14	24	14	0.98
Bevacizumab	39	23	38	23	0.98
Cetuximab	50	38	50	30	0.24
